# Levetiracetam Liver Injury: A Benign Antiepileptic Agent?

**DOI:** 10.14309/crj.0000000000001003

**Published:** 2023-03-16

**Authors:** Yassmin Hegazy, Page Axley, Goo Lee, Meagan Gray

**Affiliations:** 1 Department of Medicine, University of Alabama Birmingham Hospital, Birmingham, AL; 2 Department of Internal Medicine, University of Alabama Birmingham Hospital, Birmingham, AL; 3 Department of Gastroenterology and Hepatology, University of Alabama Birmingham Hospital, Birmingham, AL; 4 Department of Pathology, University of Alabama Birmingham Hospital, Birmingham, AL

**Keywords:** autoimmune hepatitis, levetiracetam, liver injury

## Abstract

Levetiracetam is a commonly prescribed antiepileptic agent and has rarely been linked to hepatotoxicity. This case describes a patient with drug-induced autoimmune hepatitis secondary to levetiracetam.

## INTRODUCTION

A patient with no previous liver disease or autoimmune history presented with acute liver injury consistent with drug-induced autoimmune hepatitis (AIH) in the setting of recent levetiracetam (LEV) initiation.

## CASE REPORT

**Figure 1.**
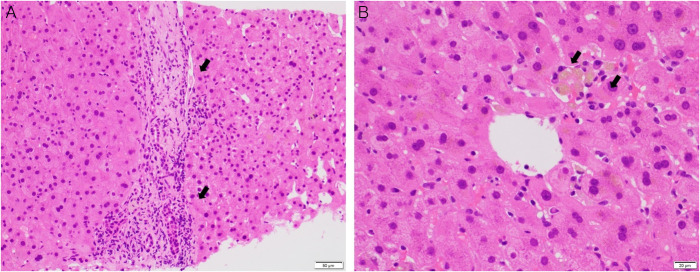
Liver biopsy findings. (A) A portal tract (arrows) showed mild inflammatory infiltrate consisting predominantly of lymphocytes with mild interface activity, bile duct injury, and bile ductular proliferation. (B) A focus of the centrilobular area demonstrated an inflammatory infiltrate with a cluster of ceroid-laden macrophages and few plasma cells (arrows).

**Figure 2.**
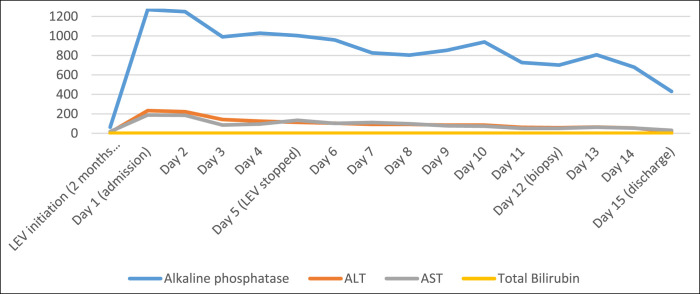
The patient's liver injury time line to levetiracetam. ALT, alanine aminotransferase; AST, aspartate aminotransferase; LEV, levetiracetam.

A 76-year-old African American woman presented with 2 weeks of worsening mental status. Her history was significant for an ischemic stroke 2 months before presentation complicated by partial seizures with initiation of LEV 1,500 mg twice daily. There were no other new medications or supplement use. On admission, her vitals were normal and physical examination revealed dry mucous membranes and orientation only to self without any new neurological deficits. Significant laboratory findings included serum sodium 161 mmol/L, creatinine 5.8 mg/dL (baseline 0.6 mg/dL), total bilirubin 0.9 mg/dL, alanine aminotransferase 233 U/L, aspartate aminotransferase (AST) 88 U/L, alkaline phosphatase 1,270 IU/L, international normalized ratio 1.28, gamma glutamyl transferase 1,442 U/L, and ammonia 352 mg/L (2 months before admission, liver function tests were normal). Serologic evaluation revealed low-level titers of autoantibodies including positive anti-nuclear antibody (ANA; 1:80), positive anti-smooth muscle antibody (ASMA; 1:80), and an elevated total immunoglobulin G at 2,162 g/L. Her anti-mitochondrial antibody was negative. Abdominal computed tomography was unremarkable. Liver biopsy revealed mild portal and lobular inflammation with mild interface activity as well as bile duct injury and bile ductular proliferation without ductopenia, steatosis, or fibrosis (Figure 1). LEV-induced AIH was diagnosed in the setting of positive autoimmune serologies and consistent liver histology. LEV was discontinued on day 5 of hospitalization, and liver tests were markedly improved by day 15 (alkaline phosphatase 430 U/L, alanine aminotransferase 16 U/L, aspartate aminotransferase 31 U/L; Figure 2). Her encephalopathy resolved with intravenous hydration and electrolyte correction. The patient was lost to follow-up with no repeat laboratory tests after discharge.

## DISCUSSION

While there have been no reported cases of AIH caused by LEV, drug-induced AIH has been reported with similar presentations to our case including the presence of positive antibodies, elevated immunoglobins, and consistent histopathology findings.^1^ AIH has been described in previous cases from antibiotics, viruses, herbal remedies, and vaccines that can trigger the immune system.^2^ Prior cases of drug-induced AIH note middle-aged, female predominance (78%) with positive ANA (78%–83%) and ASMA titers (45%–50%).^3,4^ Biopsy findings are most often identical to AIH, with lobular inflammation, plasma cell infiltration, and interface hepatitis.^5,6^

LEV has not previously been reported to cause drug-induced AIH; however, LEV has been associated with Drug Rash with Eosinophilia and Systemic Symptoms. Most patients develop Drug Rash with Eosinophilia and Systemic Symptoms within 1–5 weeks of LEV initiation, with a hepatocellular pattern of injury that resolves with discontinuation of the offending drug and steroid initiation.^7^ The pathogenesis is unclear and likely multifactorial including genetic deficiencies in drug metabolizing enzymes that can cause visceral organ inflammation including the liver.^8^ Several case reports have shown this pattern of hepatocellular liver injury related to LEV initiation and subsequent discontinuation.^9,10^

In our case, the time line of LEV initiation 2 months before presentation with significant liver injury on initial presentation in the setting of positive autoimmune serologic markers and classic biopsy findings was consistent with a diagnosis of drug-induced AIH (Figure 1 and 2). In addition, AIH can be characterized into 2 types, with both AIH type 1 and AIH type 2 common in female patients with the evidence of elevated serum immunoglobulin G and similar histopathology findings. AIH type 2 is more common in pediatric populations and characterized by the presence of anti-liver-kidney microsomal type 1 antibody. AIH type 1 is consistent with our patient's presentation given our patient's older age at onset, presence of ASMA and ANA, as well as lack of extrahepatic autoimmune disease or known inherited immune deficiency. Using the simplified scoring system by the International Autoimmune Hepatitis Group, our patient's score of 8 with definite diagnosis of AIH >7 and probable AIH score >6 also supports our AIH diagnosis based on our patient's serum globulins/IgG levels more than 1.1 the upper limit of normal, 1:80 ANA and ASMA titers, negative hepatitis viral markers, and liver histology patterns that were consistent with interface hepatitis and biliary change.^11^

While treatment of drug-induced AIH can vary, the most important treatment is discontinuation of the offending treatment, with many patients experiencing spontaneous improvement within 1 month of withdrawal. In severe symptomatic cases, corticosteroid therapy can be used, with some patients going on to need long-term immunosuppression.^3^ Corticosteroid treatment can be appropriate in some patients with hepatic failure, serum hypoalbuminemia, and/or no evidence of recovery after 1–2 weeks of cessation of the offending agent.^12^ Common long-term immunosuppression medications for patients with AIH include azathioprine with or without corticosteroid therapy as well as calcineurin inhibitors including cyclosporine and tacrolimus in refractory AIH. Other immunosuppressant medications including methotrexate and rituximab have also been effective in remission in patients with AIH. Ongoing monitoring of liver tests for at least 6 months after corticosteroid withdrawal is recommended to evaluate for relapse.^6^ In a study comparing AIH and drug-induced AIH, all patients with drug-induced AIH achieved remission of disease off immunosuppression compared with only 35% with AIH.^13^

Our case demonstrates the first reported LEV-induced AIH that resolved with prompt discontinuation of LEV. LEV-induced AIH should be considered in patients with recent LEV initiation and newly elevated liver tests. Cessation of LEV may be sufficient to resolve the liver injury without corticosteroid treatment in some cases.

## DISCLOSURES

Author contributions: Y. Hegazy: Writing the manuscript and data gathering. P. Axley: Reviewing and editing the manuscript. G. Lee: Providing pathology imaging and reviewing the manuscript. M. Gray: Reviewing the manuscript, overseeing the project, and is the article guarantor.

Financial disclosure: None to report.

Informed consent could not be obtained for this case report. All identifying information has been removed.
